# Data on short-term effect of nitrogen dioxide on cardiovascular health in Wallonia, Belgium

**DOI:** 10.1016/j.dib.2017.12.056

**Published:** 2018-01-01

**Authors:** Philippe Collart, Dominique Dubourg, Alain Levêque, Natalia Bustos Sierra, Yves Coppieters

**Affiliations:** a Centre de Recherche Epidémiologie, Biostatistiques, Recherche Clinique, School of Public Health, Université Libre de Bruxelles (U.L.B.), Route de Lennik 808, CP 596, 1070 Brussels, Belgium; b Agence Pour une Vie de Qualité, Rue de la Rivelaine 21, 6061 Charleroi, Belgium; c Institut Scientifique de Santé Publique, Santé Publique et Surveillance, Rue J. Wytsman 14, 1050 Brussels, Belgium

## Abstract

Data presented in this article are related to the research paper entitled “Short-term effects of nitrogen dioxide on hospital admissions for cardiovascular disease in Wallonia, Belgium.” (Collart et al., in press) [1].

Nitrogen dioxide concentrations showed a strong seasonal pattern with higher levels in the cold period than in the warm period. A minimum of 13.1 µg/m^3^ in July and a maximum of 26.9 µg/m^3^ in January were observed. The coldest months are December, January and February and the hottest months are June, July and August. Temperature and nitrogen dioxide were negatively correlated in the cold period and positively correlated in the warm period.

For the period 2008–2011 there were 113 147 hospital admissions for cardiovascular disease. Forty-five percent of patients were women and 66.5% were 65 and older. Heart rhythm disorders account for the majority of hospital admissions for cardiovascular disease. Our data confirms the existence of an association between NO_2_ and cardiovascular disease. Apart from haemorrhagic stroke, the strongest association between NO_2_ concentrations and number of hospital admissions is observed at lag 0. For haemorrhagic stroke, the association is strongest with a delay of 2 days. All associations calculated without stratification are statistically significant and range from an excess relative risk of 2.8% for myocardial infarction to 4.9% for haemorrhagic strokes.

**Specifications Table**

**Table:** 

Subject area	*Environmental epidemiology*
More specific subject area	*Effects of air pollution on cardiovascular health*
Type of data	*Tables and figures*
How data was acquired	*Health data were taken from a register kept by Belgian hospitals and environmental data were collected from monitoring stations.*
Data format	*Analyzed data*
Experimental factors	*Statistical analysis with R software (The R Foundation for Statistical Computing) with the mgcv and spline packages.*
Experimental features	*Generalized additive model was applied to analyse the data with a quasi-Poisson regression.*
Data source location	*Wallonia, Belgium*
Data accessibility	*Data is with this article.*

**Value of the data**

• Extensive data on temporal variations of nitrogen dioxide concentrations.
• Extensive data on the lag effect of nitrogen dioxide on different cardiovascular disease across age groups.
• The data on short-term effect of air pollution on health could be useful for government and health workers to make decisions that could reduce the risk of disease among the population.
• The result from the analysis can be compared with other environmental studies.

## 1 Data

Many studies have shown a short-term association between NO_2_ and cardiovascular disease. However, few data are available on the delay between exposure and a health-related event. The aim of the present study is to determine the strength of association between NO_2_ and cardiovascular health in Wallonia for the period 2008–2011.

## 2 Experimental design, materials and methods

### 2.1 Geographical area

The present study was conducted in Wallonia from 1 January 2008 to 31 December 2011. Wallonia is the southern region of Belgium, with an area of 16 844 km^2^ and 3 525 000 inhabitants in 2011.

### 2.2 Environmental data

Concentrations of NO_2_ and temperature for the period between 2008 and 2011 were obtained from the ISSeP (Institut scientifique de service public). Temperature data were collected from five measuring stations while NO_2_ concentrations were recorded by 10 stations spread across the study area. NO_2_ was analysed by chemoluminescence (Horiba, APEA-370). Correlation between monitoring stations was analysed using Spearman's rank correlation. More than 75% of the correlation coefficients between stations were higher than 0.75 (Table 1). The daily concentrations of each pollutant were averaged from the available results. Averaged pollution data were used as surrogates of individual exposure.

**Table 1:** Correlations between monitoring stations.

	CH01	CH03	CH04	SG01	LG03	LG06	NT03	NT04	NTO6	NT07
CH01	1	–	–	–	–	–	–	–	–	–
CH03	0.92	1	–	–	–	–	–	–	–	–
CH04	0.92	0.82	1	–	–	–	–	–	–	–
SG01	0.86	0.82	0.84	1	–	–	–	–	–	–
LG03	0.89	0.86	0.85	0.86	1	–	–	–	–	–
LG06	0.87	0.89	0.83	0.81	0.94	1	–	–	–	–
NT03	0.85	0.82	0.81	0.78	0.87	0.84	1	–	–	–
NT04	0.79	0.77	0.73	0.69	0.77	0.79	0.76	1	–	–
NT06	0.76	0.76	0.69	0.65	0.78	0.79	0.78	0.92	1	–
NT07	0.70	0.62	0.61	0.69	0.68	0.60	0.60	0.72	0.72	1

Nitrogen dioxide concentrations showed a strong seasonal pattern with higher levels in the cold period than in the warm period (Table 2). A minimum of 13.1 µg/m^3^ in July and a maximum of 26.9 µg/m^3^ in January were observed. The coldest months are December, January and February and the hottest months are June, July and August

**Table 2:** Temperature and NO_2_ concentration in Wallonia, 2008–2011.

	Percentiles					Mean ± sd
	5%	25%	50%	75%	95%	
**NO**_**2**_ (µg/m^3^)						
All year (n=1461)	8.9	13.6	18.7	25.9	36.9	20.5 ± 9.1
Warm period (n=368)	7.7	11.2	13.5	16.6	22.8	14.2 ± 4.6
Cold period (n=361)	11.1	17.3	24.8	32.9	45.4	26.1 ± 11.0
						
**Temperature** (°C)						
All year (n=1461)	-0.9	5.8	11.1	16.3	21.1	10.8 ± 6.8
Warm period (n=368)	13.4	15.8	17.7	23.4	18.0	18.0 ± 3.2
Cold period (n=361)	-3.8	0.5	3.3	6.1	9.2	3.0 ± 4.1

Abbreviations: NO_2_: nitrogen dioxide; sd: standard deviation.

Warm period: June–August; cold period: December–February.

**Table 3:** correlation between temperature and NO_2_ concentration.

	r_s_	p value
All year (n=1461)	−0.54	< 0.001
Warm period (n=368)	−0.59	< 0.001
Cold period (n=361)	0.22	< 0.001

Abbreviations: r_s_: Spearman's rank correlation coefficient

Temperature and nitrogen dioxide were negatively correlated in the cold period and positively in the warm period Table 3.

### 2.3 Hospital admissions data for cardiovascular diseases

The analyses presented below relate to patients admitted in an hospital between 25 and 104 years of age over time. The daily counts of hospital admissions for cardiovascular disease were taken from the’Résumé Hospitalier Minimum’ (RHM) for 42 hospitals within the study region. The RHM is a mandatory register kept by Belgian hospitals containing patient data (e.g.: year of birth, gender, place of residence) and stay data (e.g.: admission date). Clinical admission diagnoses were registered using the ICD-9 codes (International Classification of Disease, 9^th^ version). The daily counts of hospital admissions for CVD were graded: arrhythmia (ICD9: 426 and 427), acute myocardial infarction (AMI, ICD9: 410), ischemic stroke (ICD9: 433, 434 and 435) and haemorrhagic stroke (ICD9: 430, 431 and 432).

For the period of analysis there were 113 147 hospital admissions for cardiovascular disease (Table 4). Forty-five percent of patients were women and 66.5% were 65 and older. Heart rhythm disorders account for the majority of hospital admissions for cardiovascular disease, that is, more than 50,000 cases. The number of hospital admissions for CVD is stable during the period of analysis with 28 403, 28 284, 28 073 and 28 387 hospital admissions for 2008, 2009, 2010 and 2011 respectively. Temperature and season have little effect on the number of hospital admissions.

**Table 4:** Number of hospital admission stratified by gender, age group, season and temperature.

	CVD	Arrhythmia	AMI	Ischemic stroke	Haemorrhagic stroke
***Overall***	113 147 (100%)	52 937 (46.8%)	21 491 (19.0%)	32 902 (29.1%)	5 817 (5.1%)
					
***Gender***					
Female	50 691 (44.8%)	24 286 (45.9%)	7 110 (33.1%)	16 283 (49.5%)	3 012 (51.8%)
Male	62 456 (55.2%)	28 651 (54.1%)	14 381 (66.9%)	16 619 (50.5%)	2 805 (48.2%)
					
***Age group, year***					
25–54	17 903 (15.8%)	8 291 (15.7%)	5 136 (23.9%)	3 345 (10.2%)	1 131 (19.4%)
55–64	20 039 (17.7%)	9 177 (17.3%)	5 006 (23.3%)	4 956 (15.1%)	900 (15. 5%)
≥65	75 205 (66.5%)	35 469 (67.0%)	11 349 (52.8%)	24 601 (74.8%)	3 786 (65.1%)
					
***Season***					
Warm	27 164 (24.0%)	12 791 (24.2%)	5 017 (23.3%)	7 947 (24.2%)	1 409 (24.2%)
Cold	28 204 (24.9%)	12 919 (24.4%)	5 545 (25.8%)	8 285 (25.2%)	1 455 (25.0%)
					
***Temperature***					
High	29 577 (26.1%)	14 036 (26.5%)	5 471 (25.5%)	8 601 (26.1%)	1 469 (25.3%)
Low	29 798 (26.3%)	13 769 (26.0%)	5 747 (26.7%)	8 706 (26.5%)	1 576 (27.1%)

Warm period: June–August; cold period: December–February.

High temperature: >P75 (16.3 °C); Low temperature: <P25 (5.8 °C)

CVD: cardiovascular diseases; AMI: acute myocardial infarction.

Atrial fibrillation and flutter (ICD9 427.3) account for the majority (62.3%) of hospital admissions for heart rhythm disorders.

### 2.4 Data analysis

A time-series design was used to assess the association between short-term exposure to air pollution and hospital admissions for CVD. The generalized additive model was applied to analyse the data with a quasi-Poisson regression to account for overdispersion [2]. The day of the week was modelled using an indicator variable. Adjustment for temperature was performed with a natural spline as a smoothing function with three degrees of freedom to overcome the non-linear effect of temperature [3]. Seasonality and long-term trend were also modelled using a natural spline with three degrees of freedom per year [2], [3],[2], [3]. NO_2_ was added in the model as a linear term without a delay (lag 0) and with a delay (lag 1 to 6). The lagged variables were introduced in the model separately. The effect of the delay was analysed for the association between NO_2_ and CVD stratified over age and for the association between NO_2_ and the hospital admissions for various cardiovascular pathologies. For each subgroup analysed, the lag giving the strongest association was selected. Residuals and partial autocorrelation were checked graphically to ensure the goodness of the model. The sum of the absolute values of the partial autocorrelation function was calculated for each degree of freedom. The analyses were performed without stratification (overall analysis) and with stratification on gender and age in three subgroups: 25–54, 55–64 and ≥ 65 years. Excess relative risk (ERR) and 95% confidence intervals (CI) were calculated using a Poisson regression model and R software 2.15.0 (The R Foundation for Statistical Computing) with the mgcv and spline packages. The excesses relative risks for an increase of 10 µg/m^3^ of NO_2_ are presented in the tables.

### 2.5 Sensitivity analysis

Sensitivity analysis was performed to check the robustness of the model. Analysis using different degrees of freedom of the two natural splines was performed to estimate the effects on the strength of association. This sensitivity analysis was conducted using total daily admissions for CVD. The number of degrees of freedom giving the lowest sum of the absolute values of the partial autocorrelation function was selected.

The association of NO_2_ and AMI hospital admissions decreases slightly when the number of degrees of freedom of the smoothing function used for seasonal adjustment increases. The sum of the absolute values of the partial autocorrelation function was lowest for 3 df (Table 5).

**Table 5:** Effect of the degree of freedom of the smoothing function use to adjust for seasonality on the association between NO_2_ and CVD.

Degree of freedom per year	ERR [IC 95%]	Sum of the absolute values of PACF
1	3.6 [2.5; 4.8]	1.495
2	3.6 [2.5; 4.8]	1.468
3	3.6 [2.5; 4.8]	1.441
4	3.1 [1.9; 4.3]	1.455
5	2.3 [1.1; 3.5]	1.469
6	2.5 [1.3; 3.7]	1.555
7	2.4 [1.1; 3.6]	1.711

ERR: excess of relative risk; PACF: partial autocorrelation function.

### 2.6 Modelling results

#### 2.6.1 The lag effect

**Fig. 1:**
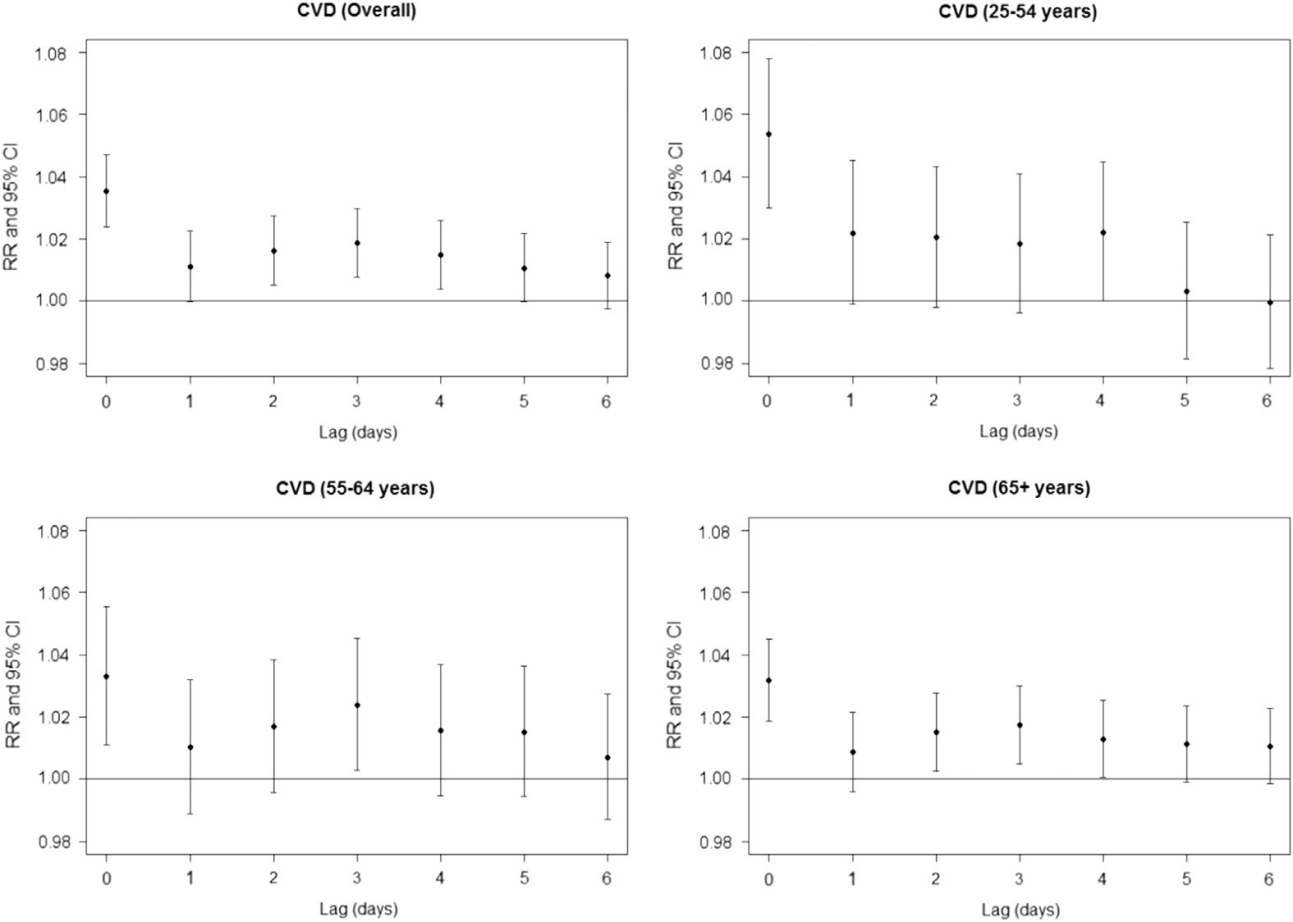
Relative risks (RR with 95% confidence intervals) for the association between NO_2_ and CVD per 10 µg/m3 increase of NO_2_ concentration obtained with the single lag model. Analysis performed for different age groups.

**Fig. 2:**
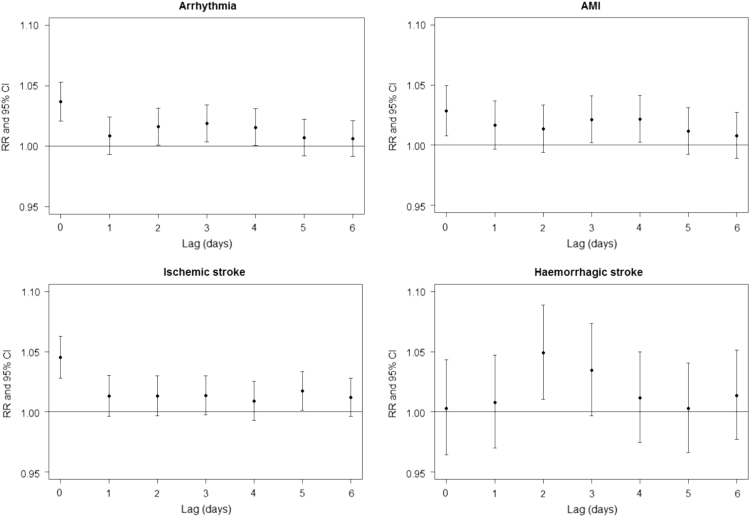
Relative risks (RR with 95% confidence intervals) for the association between NO_2_ and different cardiovascular diseases per 10 µg/m3 increase of NO_2_ concentration obtained with the single lag model. Analysis performed for all age groups.

The effect of age on the lag pattern of the association between NO_2_ concentrations and CVD is shown in Fig. 1. Regardless of age, the association is strongest taking into account the measurement of pollution on the day of the event (lag 0). The lag patterns for the four cardiovascular diseases are presented in Fig. 2. For all ages, the strongest association between NO_2_ concentrations and the number of hospital admissions is observed at lag 0 (Fig. 2), except for haemorrhagic stroke where the strongest association occurs at lag 2.

#### 2.6.2 Excess of risk

The associations between NO_2_ concentrations and the various pathologies are presented in Table 6. All associations calculated without stratification are statistically significant and range from an ERR of 2.8% for myocardial infarction to 4.9% for haemorrhagic strokes. Apart from haemorrhagic stroke where excess relative risk is 7.3% for women and 2.3% for men, gender has very little effect on the association between NO_2_ and cardiovascular disease. However, age has a more marked impact. For heart rhythm disorders and haemorrhagic stroke, extreme age groups are the most susceptible to NO_2_. For myocardial infarction, the strongest association is found for the 55–64 years age group. Temperature has a modifying effect on the association between NO_2_ and hospital admissions for CVD as well as on heart rhythm disorders and myocardial infarction. For these pathologies, the effect of NO_2_ is far more pronounced when the temperature is above 16.3 °C (P75). The season has a similar effect on CVD and heart rhythm disorders but has no effect on myocardial infarction.

**Table 6:** NO_2_ effects (risk excess and 95% confidence intervals).

Parameters	CVD (n=113 147)		Arrhythmia (n=52 937)		AMI (n=21 491)		Ischemic stroke (n = 32 902)		Haemorrhagic stroke (5 817)	
	ERR (%)	[95% IC]	ERR (%)	[95% IC]	ERR (%)	[95% IC]	ERR (%)	[95% IC]	ERR (%)	[95% IC]
***Overall***	3.5	[2.4; 4.7]	3.7	[2.1; 5.3]	2.8	[0.8; 4.9]	4.5	[2.8; 6.3]	4.9	[1.1; 8.9]
										
***Gender***										
Female	3.4	[1.9; 5.0]	3.4	[1.4; 5.6]	2.8	[−0.1; 6.4]	4.5	[2.0; 7.0]	7.3	[2.1; 12.8]
Male	3.6	[2.2; 5.1]	3.9	[1.9; 5.9]	2.9	[0.3; 5.5]	4.6	[2.3; 7.0]	2.3	[−3.2; 8.0]
										
***Age group, year***										
25–54	5.4	[3.0; 7.8]	7.6	[4.1; 11.1]	1.0	[−3.0; 5.0]	6.6	[1.1; 12.4]	3.8	[−4.6; 12.9]
55–64	3.3	[1.1; 5.5]	2.5	[−0.1; 5.8]	4.5	[0.3; 8.8]	4.6	[0.4; 9.0]	−0.1	[−9.0; 9.8]
≥65	3.2	[1.9; 4.5]	3.1	[1.3; 4.9]	3.0	[0.3; 5.8]	4.2	[2.3; 6.2]	6.5	[1.7; 11.5]
										
***Season***										
Warm	7.0	[3.1; 10.9]	11.0	[5.5; 16.7]	0.0	[−6.5; 6.9]	5.5	[−0.1;11.4]	5.2	[−7.4; 19.6]
Cold	1.8	[0.0; 3.6]	0.7	[−1.7; 3.2]	0.9	[−2.2; 4.0]	4.6	[2.0; 7.3]	1.1	[−4.6; 7.4]
										
***Temperature***										
High	6.1	[3.2; 9.2]	7.5	[3.4; 11.7]	6.2	[0.9; 11.8]	4.6	[0.2; 9.1]	2.8	[−6.9; 13.7]
Low	3.0	[1.1; 4.8]	2.2	[−0.3; 4.8]	3.0	[−0.2; 6.3]	4.7	[2.0; 7.5]	1.8	[−4.5; 5.1]

Warm period: June–August; cold period: December–February; High temperature: >P75 (16.3 °C); Low temperature: <P25 (5.8 °C).

CVD: cardiovascular diseases; AMI: acute myocardial infarction.

The analysis were performed at lag 0 for CVD, arrhythmia, AMI, Ischemic stroke and at lag 2 for Haemorrhagic stroke.
